# Pathological Insight into 5-HT_2B_ Receptor Activation in Fibrosing Interstitial Lung Diseases

**DOI:** 10.3390/ijms22010225

**Published:** 2020-12-28

**Authors:** Anna Löfdahl, Göran Tornling, Jenny Wigén, Anna-Karin Larsson-Callerfelt, Christina Wenglén, Gunilla Westergren-Thorsson

**Affiliations:** 1 Lung Biology, Department of Experimental Medical Science, Lund University, BMC C12, 22184 Lund, Sweden; jenny.wigen@med.lu.se (J.W.); anna-karin.larsson_callerfelt@med.lu.se (A.-K.L.-C.); gunilla.westergren-thorsson@med.lu.se (G.W.-T.); 2 AnaMar AB, Medicon Village, Scheeletorget 1, 22381 Lund, Sweden; christina.wenglen@anamar.com (C.W.); goran.tornling@anamar.com (G.T.); 3 Respiratory Medicine Division, Department of Medicine Solna, Karolinska Institutet, 17177 Stockholm, Sweden

**Keywords:** 5-HT, 5-HT_2B_ receptor antagonism, fibrosis, ILD

## Abstract

Interstitial lung disease (ILD) encompasses a heterogeneous group of more than 200 conditions, of which primarily idiopathic pulmonary fibrosis (IPF), idiopathic nonspecific interstitial pneumonia, hypersensitivity pneumonitis, ILD associated with autoimmune diseases and sarcoidosis may present a progressive fibrosing (PF) phenotype. Despite different aetiology and histopathological patterns, the PF-ILDs have similarities regarding disease mechanisms with self-sustaining fibrosis, which suggests that the diseases may share common pathogenetic pathways. Previous studies show an enhanced activation of serotonergic signaling in pulmonary fibrosis, and the serotonin (5-HT)_2_ receptors have been implicated to have important roles in observed profibrotic actions. Our research findings in support by others, demonstrate antifibrotic effects with 5-HT_2B_ receptor antagonists, alleviating several key events common for the fibrotic diseases such as myofibroblast differentiation and connective tissue deposition. In this review, we will address the potential role of 5-HT and in particular the 5-HT_2B_ receptors in three PF-ILDs: ILD associated with systemic sclerosis (SSc-ILD), ILD associated with rheumatoid arthritis (RA-ILD) and IPF. Highlighting the converging pathways in these diseases discloses the 5-HT_2B_ receptor as a potential disease target for PF-ILDs, which today have an urgent unmet need for therapeutic strategies.

## 1. Introduction

The term interstitial lung disease (ILD) encompasses a large heterogeneous group of diffuse parenchymal lung disorders, of which primarily idiopathic pulmonary fibrosis (IPF), idiopathic nonspecific interstitial pneumonia, ILD associated with autoimmune diseases, hypersensitivity pneumonitis and sarcoidosis may present a progressive fibrosing (PF) phenotype [1]. Despite known or unknown causes and radiological patterns, the PF-ILDs have similarities regarding disease mechanisms with self-sustaining fibrosis [2], suggesting common pathogenetic pathways. In this review, we will address the potential role of serotonin (5-HT) and the 5-HT_2B_ receptor in three PF-ILDs: IPF, ILD associated with systemic sclerosis (SSc-ILD) and ILD associated with rheumatoid arthritis (RA-ILD).

### 1.1. Idiopathic Pulmonary Fibrosis

Idiopathic pulmonary fibrosis is defined as usual interstitial pneumonia (UIP) based on high-resolution computed tomography (HRCT) and/or histopathological pattern after exclusion of other known causes of ILD [3]. IPF is the most common PF-ILD, and in a systematic review a conservative estimate of the incidence was 3–9 cases per 100,000 per year for Europe and North America with lower reports for East Asia and South America [4]. IPF is more prevalent in the older population, rarely diagnosed before the age of 70, and is more widely represented in males [5]. IPF patients demonstrate large heterogeneity in their pulmonary manifestation of fibrosis, and it is generally not regarded as an inflammatory disease, despite previous attempts to treat the disease with corticosteroids. Nonetheless, patients with a rapid progress or experiencing an acute exacerbation have reported severe innate and adaptive inflammatory infiltrates where the extent of inflammation was correlated with yearly forced vital capacity (FVC) decline [6].

It is now widely recognized that the aetiology of IPF is a gene-environment interaction involving a heterogeneous set of susceptibility genes such as *TERT*, *SFTPC*, *TOLLIP* and *MUC5B* [7]. Environmental factors that have been linked to the development of IPF in epidemiological studies include smoking, chronic viral infections and occupational exposures, such as agriculture and farming, livestock, wood dust, metal dust, stone dust and silica [8]. The clinical course of IPF is highly heterogeneous, but carries a poor prognosis with a mean survival of four years [9]. On a yearly basis 5–10% of IPF patients experience acute deteriorations in respiratory function, exacerbations, with a median survival of 3 to 4 months [10].

### 1.2. Systemic Sclerosis

Systemic sclerosis is an autoimmune disease characterized by vasculopathy of small vessels, immune dysregulation, chronic inflammation, and subsequent fibrosis of the skin and internal organs [11]. Skin fibrosis (scleroderma) is the distinguishing hallmark of SSc, and the extent of skin involvement and its rate of progression reflect the severity of visceral organ involvement. The reported prevalence of SSc varies between studies, but has been estimated to be 15–30 cases per 100,000 individuals worldwide [12], with a peak onset described at 55–69 years of age [13]. Although SSc, like other autoimmune diseases, is more common in women than in men, the male sex is a poor prognostic factor with more frequent and severe organ involvement [14].

ILD is a common and early manifestation of SSc, and most patients who develop severe restrictive lung disease do so in the first five years following the onset of SSc symptoms [15]. SSc-ILD often has a severe course, and was the leading cause of death (17%) in a large observational study in SSc [16]. The estimated prevalence of ILD has been reported at up to 84% on HRCT [17], and it has been suggested that pulmonary function tests should not be used for screening of ILD in SSc due to a lower sensitivity than HRCT [18]. The most common ILD pattern in SSc patients is nonspecific interstitial pneumonia (NSIP), although UIP can also be seen in 25–40% of cases [19].

Patients are grouped into limited cutaneous SSc (lcSSc), where the skin fibrosis is restricted to areas distal to the elbows and knees, and diffuse cutaneous SSc (dcSSc) with involvement also of the proximal extremities and trunk. The extent of skin involvement is a prognostic risk factor for ILD and patients with dcSSc have both a higher prevalence and mortality from ILD than those with lcSSc [16,20]. In addition, male sex, ethnicity and presence of anti-topoisomerase antibodies appear to be important determinants of ILD development [17].

### 1.3. Rheumatoid Arthritis

Rheumatoid arthritis is a systemic inflammatory autoimmune disorder estimated to affect 0.5–1% of the world’s population. Although the predominant clinical feature is inflammation of the synovial lining of joints, RA has numerous extra-articular manifestations, and lung disease is a major contributor to the extra-articular morbidity and mortality. There are strong indications that lungs are involved in early pathogenesis of RA by citrullination of proteins triggered by environmental exposure of, e.g., tobacco smoke. Development of anti-citrullinated protein antibodies (ACPAs) in genetically susceptible individuals [21] may initiate inflammatory responses and autoimmune reactivity. ILD is one of the most common comorbidities associated with RA, significantly aggravating the patient’s disease course, prognosis, and health-related quality of life [22]. The prevalence of RA-ILD has been reported to be as high as 76% in imaging studies, but clinically significant ILD occurs in less than 10%, albeit with increasing incidence [23].

The histopathology of RA-ILD is heterogeneous showing a highly variable mix of both fibrotic and inflammatory changes. Contrary to ILD associated with other connective tissue diseases, the most prevalent pattern in RA-ILD is UIP followed by NSIP, which can be further broken down into inflammatory and fibrotic subtypes [24]. Compared to RA patients with a non-UIP pattern, those with UIP confer a poorer prognosis with survival rates that are in parallel to those seen in IPF. The RA-ILD patients with UIP have been reported to have more respiratory-related hospitalizations than other ILD subtypes [25].

Risk factors for the development of RA-ILD have been identified in several studies and include older age, male sex, cigarette smoking, later onset RA, longer RA duration, RA disease activity, and elevated levels of rheumatoid factor or anti-ACPA [26].

### 1.4. Current Therapeutic Strategies

Treatment of PF-ILD has changed considerably during the last two decades. Corticosteroids were during many years widely used for the treatment of fibrotic lung diseases, but serious concerns have been raised due to increased mortality of IPF patients receiving prednisone, azathioprine and N-acetylcysteine in a clinical trial [27] and the risk for renal crisis in patients with SSc [28]. In patients with SSc and RA, the therapy for the underlying disease has formed the basis for treatment of the ILD component. In RA and SSc, immunosuppressive therapies including cyclophosphamide, azathioprine, and mycophenolate mofetil are widely used, and in SSc, haematopoietic stem cell transplantation has also been successful. The first targeted antifibrotic drug, pirfenidone, was introduced for the treatment of IPF in Japan 2008, and a couple of years later in the EU and the US. Another antifibrotic drug, nintedanib, was approved for treatment of IPF in the US 2014 and in the EU 2015. Since then, both drugs have been investigated in clinical studies enrolling a wide range of PF-ILDs, leading to approvals for use in SSc-ILD regarding nintedanib in both the US and in the EU, and for pirfenidone in the US. Nintedanib was further approved in 2020 in the EU for use in other chronic fibrosing ILDs with a progressive phenotype. Several antifibrotic drugs are currently investigated for IPF in clinical phase 2–3 trials, some of them also targeting a broader spectrum of PF-ILDs [29]. Today, there is no curative treatment available for ILDs where lung transplantation stands as the final therapeutic measure.

## 2. The Serotonergic Pathways in Tissue Repair and Fibrosis

Serotonin (5-hydroxytryptamine, 5-HT) is a multifunctional signaling molecule, mainly recognized for its role in the central nervous system (CNS), where it regulates several behavioral processes. Even now, over 70 years after its discovery, the functional role of 5-HT is still not fully clarified, with emerging studies showing new biological influences and disease associations. A mechanistic link between fibrosis and 5-HT was first reported in the 1960s for a condition called carcinoid syndrome which is caused by neuroendocrine carcinoid tumors that secrete vast quantities of 5-HT [30]. The syndrome was characterized by tissue fibrosis, particularly affecting cardiac valves but also impacting on other organs including lung and skin. More recently, agonistic activity on the 5-HT_2B_ receptor has been implicated in causing fibrosis, which led to the recall of fenfluramine used in the treatment of obesity, as well as pergolide, a drug used to treat Parkinson’s disease [31,32]. The 5-HT_2B_ receptor agonistic activity of these drugs has been suggested to lead to myofibroblast activation in a transforming growth factor (TGF)-β1 dependent manner, resulting in fibrosis [33,34]. Besides the 5-HT_2B_ receptor, the receptor subtypes 5-HT_2A_ and 5-HT_2C_ have also been suggested to be involved in fibrosis. 5-HT has been described to play a role in alveolar macrophage function through 5-HT_2C_ receptors and thereby affect fibrosis development [35], while the 5-HT_2A_ receptor has been shown to induce a TGF-β dependent fibrotic response in vivo [36]. Among the other classes of receptors, 5-HT_7_ was in a recent paper by Tawfik et al. suggested to mediate anti-inflammatory and anti-fibrotic effects in the bleomycin-induced lung fibrosis model in rats [37]. However, the cellular mechanisms underlying PF-ILDs are still under investigation where the activation of specific 5-HT receptors remains an overlooked target in pulmonary fibrotic disorders. To understand the pathophysiological impact of 5-HT and the different 5-HT receptors, it is important to take into account the cellular context and the diversity in expression profile of the 5-HT receptors in different conditions. It is clear that activation of the 5-HT_2B_ receptor critically affects several profibrotic responses, whereby modulating its activity has been shown to attenuate fibrosis [34,38,39,40].

### 5-HT Synthesis and Signaling

5-HT is synthesized from the amino acid L-tryptophan, which is either incorporated into newly synthesized proteins or undergoes metabolism via two pathways of rate-limiting enzymes: tryptophan hydroxylase (TPH) or indoleamine 2,3-dioxygenase (IDO) and tryptophan 2,3-dioxygenase (TDO) [41]. Following an initial hydroxylation by TPH and decarboxylation, L- tryptophan is converted to 5-HT. There are two isoforms of TPH: TPH1, expressed in neural cells and enterochromaffin cells in the gastrointestinal (GI) tract; and TPH2, expressed predominantly in the CNS. The major source of 5-HT is found outside the nervous system, synthesized by the enterochromaffin cells. Upon secretion from the GI tract, 5-HT is rapidly taken up by circulating platelets via the serotonin re-uptake transporter (SERT) and stored in dense granules. Pulmonary sources of 5-HT reside in platelet-derived 5-HT as well as in endothelial cells, mast cells and pulmonary neuroendocrine cells, which harbor and produce 5-HT [42,43].

**Figure 1:**
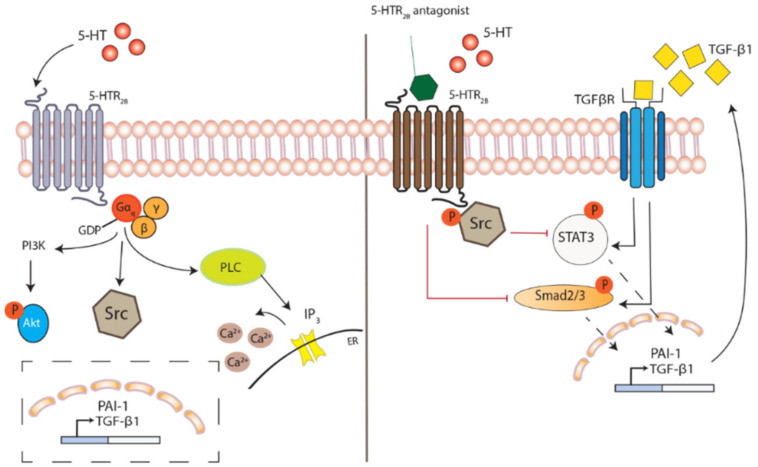
5-HT_2B_ receptor activation and inhibition. The binding of 5-HT to the G-protein coupled receptor 5-HT_2B_ (5-HTR_2B_) triggers a GDP-GTP exchange with the dissociation of α and βγ subunits, followed by activations of downstream effector molecules; phosphoinositide 3-kinase (PI3K), Src and phospholipase C (PLC). Subsequently, the transcription of plasminogen activator inhibitor (PAI)-1 and TGF-β1 become increased, activating fibrotic responses. The inhibition of receptor activation with 5-HT_2B_ receptor antagonist causes sequestering of phosphorylated Src, preventing the downstream activation and nuclear translocation of STAT3 and Smad2/3, with decreased expression of PAI-1 and TGF-β1. Conceivably, 5-HT_2B_ receptor antagonism may further reduce TGF-β receptor signaling through a diminished availability of TGF-β, thus acting as a second messenger to 5-HT.

The functions of 5-HT are mediated by binding to its receptors, where at least 15 different types of 5-HT receptors have been identified in human, all with specific tissue distributions and signaling mechanisms. The receptors are categorized into seven classes, where the class 2 receptors are subdivided into 2A, 2B and 2C and are G-protein coupled receptors (GPCR). Through the binding of 5-HT, the 5-HT_2B_ receptor activates and propagates the ligand-receptor signal by interacting with intracellular effector proteins such as phospholipase C (PLC) and inositol 1,4,5-trisphosphate (IP_3_), which ultimately trigger intracellular Ca^2+^ release. The elevated Ca^2+^ levels regulate gene expression and influence cellular responses [44] (Figure 1).

## 3. TGF-β—A Potential Second Messenger to 5-HT

TGF-β1 is recognized as a central mediator of fibrotic signaling and is secreted in an inactive form by monocytes, lymphocytes, fibroblasts and macrophages and is stored in a latent form in the extracellular matrix (ECM) [45]. Upon activation, TGF- β1 binds to cell-surface receptors and activates both the non-canonical and canonical (Smad-dependent) signaling pathways, where the latter includes translocation of proteins to the nucleus with sequential targeted gene transcription of profibrotic genes such as plasminogen activator inhibitor (PAI)-1, collagen and fibronectin. Fibroblasts from patients with SSc have shown an increased expression of TGF-β1 receptors [46] as well as cell-surface integrins, which can increase the amount of active TGF-β1 from the ECM. A small ECM component, the proteoglycan decorin, has been suggested to inhibit TGF-β activation with promising attenuated effects on fibrosis in vivo [47]. However, a dual involvement of decorin in fibrosis is described, as decorin is also shown to enhance fibroblast migration [48]. In an in vivo model of experimentally induced lung fibrosis in mice, pulmonary expression of decorin increased, which was diminished following treatment with 5-HT_2B_ receptor antagonists [34]. This oral, preventive treatment with 5-HT_2B_ receptor antagonist in bleomycin-treated mice resulted in an attenuated fibrotic development in the lung with reduced deposition of connective tissue. This phenomenon was further identified in vitro, in human lung fibroblasts, where inhibition of 5-HT_2B_ receptors resulted in reduced synthesis of total amount of proteoglycans and in particular decorin in cells exposed to 5-HT and TGF-β1 [34]. Our previous study also showed that 5-HT_2B_ receptor antagonist promotes an antiproliferative effect on human bronchial smooth muscle cells and the inhibition of TGF-β1 release [49]. The 5-HT_2B_ receptor antagonism also hampered myofibroblast differentiation as seen with reduced pulmonary count of myofibroblasts in bleomycin-treated mice, a response that appeared to be generated by interfering with TGF-β1 [34]. Significant anti-fibrotic effects with, e.g., reduced ECM production have also been observed in in vivo disease models of SSc after therapeutic treatment with selective 5-HT_2B_ receptor antagonists [50,51]. These data imply a direct or indirect link between serotonergic signaling and TGF-β1 activity, where the mediators together drive important fibrotic remodeling processes. 

### 3.1. A Piece of PAI?

The evidential impact of TGF-β1 on the development of fibrosis has been described in multiple studies, where its sole pathway activation suffices the establishment of tissue fibrosis. A study by Sonnylal et al. showed that constitutive activation of TGF-β1 signaling in fibroblasts in mice developed histopathological features of dermal fibrosis as recognized in patients with SSc [52]. The increased TGF-β1 expression was associated with vascular changes, showing thickened vascular walls, along with enhanced levels of downstream targets, such as collagen type I, fibronectin as well as PAI-1 [53]. In fibrotic tissue, the increased levels of PAI-1 influenced ECM turnover; however, its impact in disease is not yet fully understood, with studies showing both pro- and anti-inflammatory properties [53]. Lung fibroblasts from IPF patients and bleomycin-treated mice have demonstrated lower expression of PAI-1 in comparison to normal fibroblasts, with elevated levels of collagen type I and alpha-smooth muscle actin (α-SMA) in the IPF-derived fibroblasts [54]. However, other studies further support the profibrotic effect elicited by PAI, where PAI-deficient mice showed an enhanced fibroblast apoptosis with reduced myofibroblast formation [55]. The role of PAI may be linked to its early role in the onset of scarring, as mice subjected to skin injury showed a swift increase in PAI-1 expression [56]. Neutralization of PAI-1 using a monoclonal antibody administered intraperitoneally both at induction of disease and at disease establishment in a model of graft-versus-host disease, improved the clinical skin condition showing normalization of cell infiltrations, epidermal thickening and ulcer formation [57]. Additionally, the alleviating effects of PAI-1 neutralization were also demonstrated in a bleomycin model of progressive skin fibrosis [57]. Moreover, in bleomycin-injured mice, PAI-1 stimulated apoptosis of alveolar epithelial cells [58], which are regarded by many researchers as the primary cell type affected in the repeated damages manifested in the onset of IPF [59,60,61]. Thus, diminishing the expression of PAI-1 may serve as a beneficial anti-fibrotic strategy [62] that may partially protect against the development of pulmonary fibrosis.

### 3.2. Impacts of 5-HT_2_ Receptor Activation on Downstream Signaling

Several studies support the notion that 5-HT induces TGF-β and PAI-1 [38,63]. In fibroblasts isolated from patients with SSc, 5-HT has shown a dose-dependent increase in mRNA levels of TGF-β and PAI-1 [38]. 5-HT_2_ receptor antagonists have been suggested to attenuate lung fibrosis by reducing TGF-β signaling measured, e.g., as reduced Smad2/3 phosphorylation. This was recently shown in a chronic graft-versus-host disease model where inhibition of the 5-HT_2B_ receptor using a highly selective antagonist resulted in reduced dermal fibrosis and lung fibrosis as well as a decreased Smad 2/3 phosphorylation, suggesting TGF-β involvement [50] (Figure 1). The signaling pathways elicited by 5-HT_2_ receptor activation is not yet elucidated in full context, but it has been speculated that 5-HT-induced profibrotic responses are partly mediated by a second messenger. As 5-HT is known to regulate TGF-β production, TGF-β has been suggested to be this second messenger. Supporting this, Dees et al. showed a time-dependent increase in nuclear levels of p-Smad3, in response to 5-HT induction using dermal fibroblasts isolated from SSc patients [38]. Furthermore, by using neutralizing antibodies against TGF-β they showed that the profibrotic effects of 5-HT were TGF-β dependent. The antibodies completely abrogated the profibrotic effects of 5-HT on mRNA expression of collagen and fibronectin. In contrast to this, Chaturvedi et al. demonstrated that 5-HT-dependent TGF-β1 signaling activated both canonical (Smad dependent) and non-canonical signaling pathways and that 5-HT_2B_ receptor antagonists primarily affected the non-canonical pathways, ERK1/2 and STAT3 [63]. Following treatment with 5-HT_2A_ receptor antagonists, the downstream mediators of TGF-β1 pathway were shown to be affected with reduced expression of pSmad3 and pERK1/2 [64]. However, with the 5-HT_2B_ receptor antagonist (SB204741), the non-canonical pathway of TGF-β1 signaling was more influenced with physical and functional restriction of p-Src [63,65]. This proposed mechanism of 5-HT_2B_ receptor antagonists, studied in human dermal fibroblasts and porcine interstitial cells of the aortic valve, suggests that the sequestering of p-Src sequentially inhibits STAT3 phosphorylation [63,65]. Phosphorylated STAT3 signaling is overactivated in SSc patients with accumulation of p-STAT3 in the fibrotic skin. The pathological link of STAT3 in SSc is further recognized and localized to dermal fibroblasts, where STAT3 deficiency results in cellular desensitization for profibrotic effects triggered by TGF-β. Additionally, treating bleomycin-challenged mice with a STAT3-inhibitor ameliorated induced skin fibrosis [66].

Strong evidence points towards a joint activated pathway in tissue fibrosis driven by an overactivated TGF-β response, partly governed by the 5-HT_2B_ receptor. Several studies have identified 5-HT as a mediator potentiating TGF-β1-induced myofibroblast differentiation and tumor necrosis factor-alpha (TNF-α)-induced matrix mineralization [34,67]. Prevention of receptor activation with 5-HT_2B_ receptor antagonist steers intracellular signaling pathways, via STAT signaling, reduced TGF-β production and directly or indirectly via reduced Smad signaling, toward a minimized profibrotic cellular activity with alleviated ECM deposition and myofibroblast differentiation.

## 4. Vascular Impact in ILD—A Local Delivery System for 5-HT

The wound healing response requires precise temporal instructions to promote a proper assembly of cells and ECM components to restore healthy functional tissue. The initial phase following tissue injury initiates the systemic recruitment of circulating blood platelets to exposed subendothelial ECM proteins with the release of important wound mediators, e.g., TGF-β1, PAI-1, fibrinogen, platelet-derived growth factor (PDGF) and vascular endothelial growth factor (VEGF). Released from dense granules in platelets, 5-HT promotes coagulation together with tissue factor and ADP, acting as a helper agonist [68,69]. The mixture of naturally derived growth factors from activated platelets has been used in therapeutic purposes in the form of a platelet gel or as platelet-rich plasma, showing beneficial clinical effects, enhancing wound closure in patients with cutaneous ulcers [70,71]. The cascade of locally released mediators such as the aforementioned agents, along with PDGF, interleukin (IL)-1β [72], act in synergy to enhance the repair response, triggering the synthesis of ECM proteins, angiogenesis and inflammation.

The serotonergic impact of the lung is further emphasized by the local enrichment of platelets that are readily translocated and accumulated in the lung following systemic exposure of 5-HT [73]. Additionally, the lung has also been suggested to be a major site of platelet production, where intravascular megakaryocytes in the pulmonary circulation release platelets [74]. As seen in mice, the lung acts as a platelet reservoir as megakaryocytes are found in the pulmonary extravascular space [74]. This extrapulmonary source of platelets, with bone marrow-derived hematopoietic stem cells, participates in the local inflammatory process following lung injury. It has been shown that by blocking the serotonergic signaling with ketanserin (a 5-HT_2A/2C_ receptor antagonist) the inflammation and the fibrotic deposition of connective tissue in the lungs of bleomycin-treated mice were reduced along with altered pulmonary levels of hemopoietic stem and progenitor cells [75].

In general, dysregulated endothelial permeability and vascular leakage are common features in ILDs [38,76]. In SSc, there is a progressive loss of capillaries due to microvascular injury, which results in tissue fibrosis [77]. Endothelial dysfunction and vasculopathy develop early in SSc, with Raynaud’s phenomenon as a typical vascular manifestation [78,79]. In patients with RA-ILD, there is an ongoing systemic inflammation with increased risk of pulmonary hypertension, and there is a general overall risk of pulmonary thromboembolism in patients with RA [80]. Deficiency in peripheral 5-HT has been shown to exacerbate the clinical and pathological scores of arthritis in collagen-induced arthritic mice, where depletion of 5-HT in *Tph1*^-/-^ mice showed an altered inflammatory response with a cell imbalance in Th17 cells and T-regulatory cells [81]. These studies demonstrate the active role of 5-HT in autoimmunity and the important function of circulating platelets in the pathogenesis of rheumatic diseases [82].

Patients with IPF show that heterogenic abnormal vascular phenotypes with anastomoses between the systemic and pulmonary vasculature, neovascularization in fibrotic areas and secondary pulmonary hypertension are commonly occurring [83,84]. Enhanced vascularization is evident close to fibrotic areas, whereas within the fibrotic foci there is substantially reduced vascularization [85], suggesting that the surrounding cells are trying to compensate the lack of sufficient oxygen supply. Interestingly, 5-HTRs have been implied to regulate hypoxic responses in pulmonary vascular systems in pulmonary arterial hypertension (PAH) [86]. Pulmonary hypertension is a disease with vascular remodeling with features of inflammation and fibrosis and has been described as a common comorbidity in several ILDs [87]. In these vascular structures, the endothelial expression of TPH1 is increased causing serotoninergic-induced proliferation of the underlying smooth muscle layer [88]. Inhibition of TPH1 showed beneficial effects in models of PAH reducing vascular remodeling [89]. Interestingly, the 5-HT_2B_ receptor antagonist SB204741 has been shown to prevent the onset of the heritable form of PAH in vivo [90], with signs of reduced arteriole wall stiffness. The 5-HT_2B_ receptor is broadly expressed in the cardiovascular system and a pathological connection of 5-HT to cardiovascular events has been described in which therapeutic effects were obtained when serotonergic signaling was blocked in pulmonary hypertension [91].

In conclusion, there is a close cellular crosstalk in the alveolar compartments of ILDs where vascular changes in blood flow, local hypoxia and platelet activation with release of 5-HT may be a trigger for local injury and further development of fibrotic events involving 5-HT signaling and receptor activation.

## 5. The Immune Modulating Impact of 5-HT

Accumulating evidence points to the role of 5-HT as a potent immune modulator affecting various immune cells through its receptors [92] and via the process of serotonylation [93]. Almost all immune cells express the 5-HT receptors, including the 5-HT_2B_ receptor. In acute inflammation, 5-HT is believed to recruit immune cells to the inflammatory site. Exactly how 5-HT interacts with the immune system is less well characterized, but deregulated 5-HT levels have been suggested to contribute to the pathology of chronic inflammatory disorders by homing cells to the inflammatory site and to target e.g., T-cells and macrophages. M2 macrophages are strongly implicated in the pathogenesis of fibrosis, as they are a rich source in providing profibrotic mediators, highlighting the significance in blocking the polarization of M1 to M2 macrophages. In line with this, 5-HT has been shown to skew macrophage polarization [94] through engagement of the 5-HT_2B_ and 5-HT_7_ receptors and modulate T-cell activation, proliferation and differentiation and thereby cytokine production [92]. 5-HT has been shown to regulate the production of IL-1, IL-6 and TNF-α from human monocytes [95], an event also shown in mice [96]. Recently, prophylactic treatment with 5-HT_2B_ receptor antagonist resulted in reduced production of the pro-inflammatory cytokines TNF-α and IL-1β, demonstrated in a bleomycin-induced lung fibrosis model [34]. Furthermore, a report studying the anti-inflammatory potential of a 5-HT_2B_ receptor antagonist in vivo and in vitro suggested that selective inhibition of the 5-HT_2B_ receptor reduces both T cell-dependent and T cell-independent inflammatory responses [97]. Importantly, the mechanism by which the 5-HT_2B_ receptor activity controls immunological effects is inconclusive with studies supporting different pathways [98,99,100].

Alterations in 5-HT signaling have been described in inflammatory conditions of the gut, such as inflammatory bowel disease, in patients with allergic airway inflammation, RA and SSc [92]. Interestingly, a genetic polymorphism of the 5-HT_2A_ receptor has been associated with increased susceptibility to RA [101]. A direct link between systemic 5-HT levels in seropositive RA patients (the most common form of RA) and joint pain has been suggested [102], which has been confirmed in models of arthritis, where increased intra-articular levels of 5-HT caused joint inflammation and pain [103], while its depletion attenuated disease severity [104]. The amplified vascular permeability in inflamed joints suggest platelet-derived 5-HT to mediate the effect [105]. Radiographic changes in temporomandibular joints of patients with RA was associated with high 5-HT levels [106], which was also associated with synovial plasma extravasation by the release of various inflammatory mediators [107,108,109]. Furthermore, platelets of RA patients have a lower 5-HT content, which is interpreted as platelet release of 5-HT during inflammatory episodes [110], a finding that was also observed in SSc patients [111].

The immune modulating properties of 5-HT and 5-HT_2B_ receptor antagonists may thus have a beneficial effect in treating PF-ILDs, since chronic inflammatory processes and enhanced release of proinflammatory mediators contribute to tissue destruction and reconstruction, as seen in patients with SSc or RA [112,113]. Although the contribution of inflammation in fibrosis pathology is still under debate, and in IPF, little effect has been shown with corticoid steroid treatment, a disease lacking requirements of inflammatory infiltrates at diagnosis. Even so, IPF patients with a rapid deterioration display an increased infiltration of inflammatory cells [6]. As such, fibroblast derived from IPF patients cultured on plastic release enhanced levels of TNF-α promoting cellular detachment and cellular migration [114]. It is possible that inflammation may occur and have a more pronounced impact in early phases in IPF since the condition is thought to arise from repeated damage to epithelial cells with subsequent triggered inflammatory response. Due to the insidious nature of the PF-ILDs, diagnosis and pharmacological treatment are commonly initiated at a late stage when fibrosis has manifested for several years, possibly hindering therapeutic effects. Conceivably, an earlier and more targeted anti-inflammatory treatment may be required to generate measurable effects in fibrotic development, where the immune modulating impact of 5-HT_2_ receptor antagonism is yet to be elucidated. 

## 6. The Perfect Interstitial Storm—Vascular System, Inflammation and Fibrosis

### 6.1. 5-HT—From Circulation to Local Tissue Delivery

Increased systemic levels of 5-HT contribute to tissue remodeling processes, since it is swiftly delivered to damaged sites via recruited platelets. Levels of 5-HT have shown to be increased during fibrosis [36], probably linked to an enhanced platelet degranulation at injured sites. Already in 1983, platelets were shown to be depleted in 5-HT in patients with inflammatory arthritic diseases such as SSc, systemic lupus erythematosus and RA, indicative of enhanced platelet activity in these chronic disease states [110]. Circulating endothelial cells have been shown to be increased in IPF patients, particularly in those with low diffusion capacity [115], along with shredded microparticles from endothelial cells [116]. These microparticles were shown to induce fibroblast migration in vitro, which indicates that an activated endothelium may influence fibrogenesis [116].

In SSc, platelets are either directly or indirectly involved in all three pathophysiologic processes (autoimmunity-vasculopathy-fibrosis). Vasculopathy and endothelial dysfunction are early events in the pathogenesis of SSc, recognized by Raynaud’s atypical vascular manifestation presented years prior to any other symptom. In patients with Raynaud’s phenomenon [117], elevated plasma levels of 5-HT were shown to be correlated with markers for endothelial damage, e.g., von Willebrand factor (vWF), and tissue-plasminogen activator [118]. An enhanced platelet activation caused by the vasculopathy may lead to local release of 5-HT, where 5-HT may act on nearby fibroblasts to trigger fibrosis. This pathogenic feature can explain why skin fibrosis in SSc always starts from the fingers in the form of sclerodactyly as the fingers are the first sites affected by vasculopathy [78].

In bleomycin-induced pulmonary fibrosis in rats, 5-HT homeostasis was affected with increased gene expression of *Tph1* along with downregulation of *Sert* [119]. In line with this, inhibition of IDO in fibroblasts enhanced the gene expression of *Tph1*, along with elevated levels of melatonin, the secondary catabolic product of 5-HT, pointing towards a crosstalk between IDO and TPH pathways. Treatment with melatonin decreased the gene expression of IDO in fibroblasts [120]. Collectively, these findings indicate that an altered pathway activity may further propagate a specific signaling cascade and cellular response, that in fibrosis may be skewed towards over activated TPH1 and resulting in an increased production of 5-HT. Inhibition of 5-HT uptake into circulating platelets may thus reduce the systemic level and local tissue delivery of 5-HT, sequentially minimizing local inflammatory and profibrotic actions.

### 6.2. The Distribution of 5-HT_2_ Receptors—Tuning Inflammation and Fibrosis

In normal human pulmonary conditions, levels of 5-HT are usually low due to high pulmonary expression of *SERT* [121], with 5-HT_2A_ and 5-HT_2B_ receptors found on bronchial and vascular SMCs, and on endothelial cells, respectively [39]. However, in the lungs of IPF patients, the 5-HT_2A_ receptors have also been described to be localized on interstitial fibroblasts. Expression of 5-HT_2B_ receptors is mainly localized to fibroblasts in fibroblastic foci, as compared to 5-HT_2A_ receptors, and in areas of fibrotic tissue [36]. Histological examination of lung biopsies from SSc patients revealed intertwined patterns of inflammation and fibrosis; however, despite the high prevalence of pulmonary involvement, the pathogenesis of SSc-ILD is not well understood. The mesenchymal involvement is however evident with activated fibroblasts and myofibroblasts providing high amounts of deposited collagens. The 5-HT_2B_ receptor has also been repeatedly identified in the fibrotic skin of SSc patients, demonstrating an increased receptor expression with an evident localization to fibroblasts [38,122].

**Figure 2:**
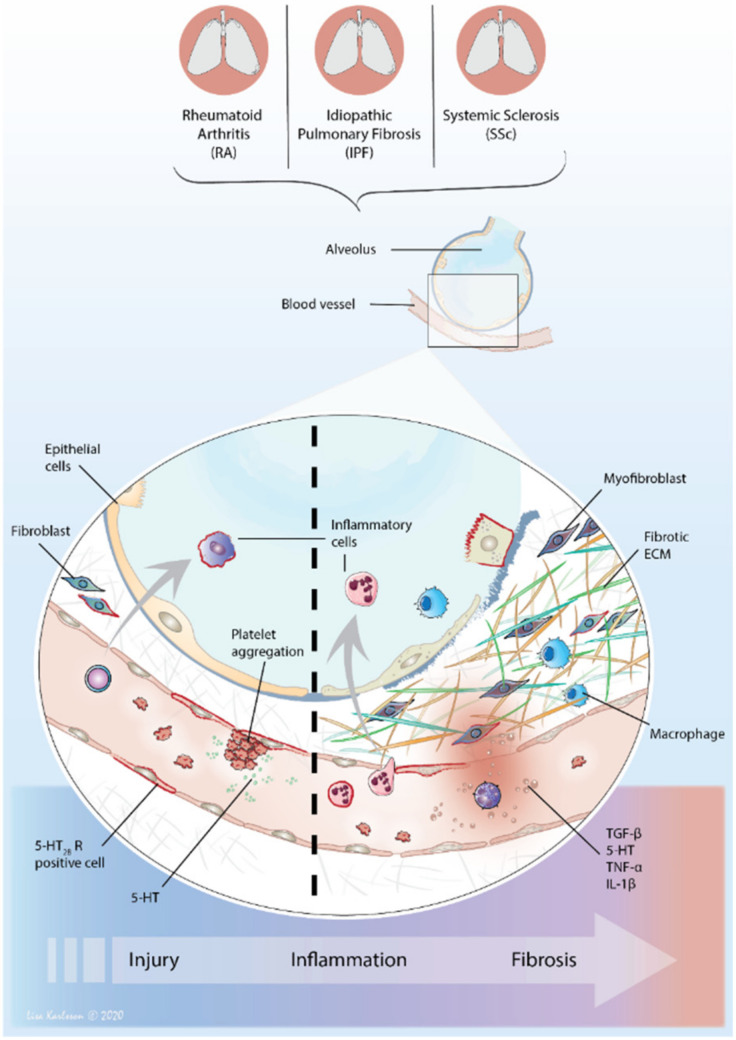
Converging pathways in PF-ILDs. The PF-ILDs of RA, IPF or SSc share similarities in disease mechanisms, where several pathogenic pathways are likely intertwined and linked to the development of lung fibrosis. At tissue injury, circulating platelets become recruited to the injured site where they aggregate and become activated, releasing 5-HT. The increased local concentration of 5-HT allows for binding to 5-HT_2B_ receptors expressed on nearby cells (outlined in red), promoting pro-inflammatory and fibrotic actions with increased permeability and release of cytokines as a result. The activation of 5-HT_2B_ receptor triggers fibroblast proliferation and differentiation into myofibroblast causing an excess deposition of ECM proteins that propels the tissue into a fibrotic state. Macrophages become polarized toward an M2-phenotype that further enhance the repair mechanism which is exaggerated in fibrosis.

Collectively, more studies align with the notion of a pathological link between altered local and systemic levels of 5-HT in diseases characterized with endothelial involvement and wound healing responses provided by fibroblasts. The role of 5-HT and its receptors in PF-ILDs such as SSc-ILD, RA-ILD and IPF has not been considerably studied, with major examinations of its potential as a novel therapeutic target still lacking (Figure 2).

### 6.3. 5-HT_2B_ Receptor—An Important Player in Fibrosis

In recent years, several studies have demonstrated potent fibrotic effects elicited through 5-HT signaling, stimulating several cellular processes that are associated with the development of fibrosis. The systemic levels of 5-HT have been shown to have marked effects on dermal fibrosis, where reduced blood levels of 5-HT resulted in protective effects against fibrotic manifestation in skin [38]. In a systemically induced experimental model of lung fibrosis, mice subjected to repeated subcutaneous administrations of bleomycin demonstrated an attenuated fibrotic development in the lung with reduced deposition of connective tissue, following an oral, preventive treatment with 5-HT_2B_ receptor antagonist [34]. Taken together, 5-HT-associated signaling is a promising target in regulating several profibrotic cellular responses in multiple organs.

In light of the presented studies examining the pathogenic impact of 5-HT with aggravated fibrosis, cytokine release and cell infiltration, one could ask whether the main antifibrotic mechanism elicited by abrogated 5-HT signaling is facilitated through its anti-inflammatory properties. However, treatment with 5-HT_2_ receptor antagonist also shows beneficial antifibrotic effects when administered in animal models with established fibrosis lacking inflammatory features [123,124,125]. The tight skin 1 mouse model (Tsk-1), reflecting human SSc, generates autoantibody production and skin fibrosis with only minor inflammation, thus modelling the disease at later stages. Treatment of Tsk-1 mice with 5-HT_2B_ receptor antagonists attenuated fibrotic development where dermal thickness, collagen deposition and myofibroblast count were reduced [51].

## 7. Therapeutic Potential in 5-HT_2B_ Receptor Antagonism

Current antifibrotic therapies reduce the rate of disease progression, defined as decline in lung function, by about 50% in IPF, as well as in other PF-ILDs. This indicates that the pathways affected by these drugs are shared across a spectrum of fibrotic lung diseases. Interestingly, the proportion of patients whose diseases are stabilized over a year has increased with these therapies; however, some treated patients still progress at a rate similar to the natural course of the disease. Thus, it is tempting to speculate that there are several endotypes of fibrosis in the spectrum of PF-ILDs, and that several pathways may be involved in a single patient. A better understanding and characterization of these endotypes with biomarkers may therefore sharpen individualized treatment, either as monotherapy, targeting a specific pathway, or as combination therapy, targeting several pathways. The need for improved characterization of patients to develop individualized therapies is evolving and is discussed further in [126,127,128].

The 5-HT_2B_ receptor represents a promising target for new anti-fibrotic treatments. The development of new drugs selectively targeting the 5-HT_2B_ receptor has so far been hampered by non-selective compounds with unwanted side effects. New, safe and highly selective 5-HT_2B_ receptor antagonists are therefore needed, and are currently in development. Using receptor antagonists to selectively block binding of 5-HT to specific receptors offers beneficial outcomes as a therapeutic strategy with minimized secondary effects in comparison to systemically reducing 5-HT levels. Clopidogrel inhibits ADP-dependent platelet activation and has shown therapeutic effects in reducing fibrosis [129,130]. It reduces the degranulation of platelets and thus influences systemic and local levels of 5-HT. However, in a small study in patients with SSc, clopidogrel did not reduce freely circulating levels of 5-HT in plasma and showed no effect in reducing dermal thickening.

**Figure 3:**
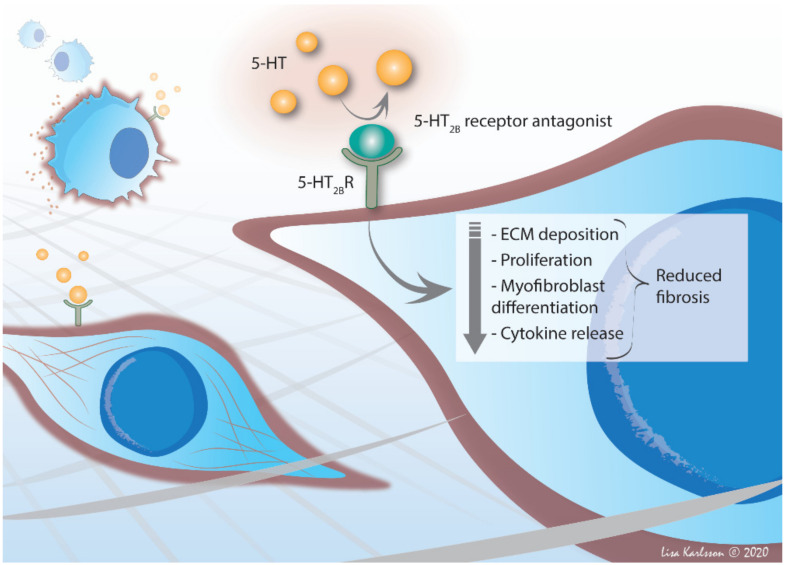
Potential of 5-HT_2B_ receptor antagonism in PF-ILDs. By blocking the binding of 5-HT with selective 5-HT_2B_ receptor antagonist, several key events in fibrosis can be inhibited. This as-yet unexplored therapeutic strategy has been demonstrated in pre-clinical models to reduce, e.g., ECM deposition, cell proliferation, myofibroblast differentiation and proinflammatory cytokine release, ultimately attenuating the development of lung fibrosis, an effect also observed in other types of tissue, further emphasizing 5-HT_2B_ receptor as a novel disease target for anti-fibrotic treatment.

With a central role in fibrosis, TGF-β has been suggested to be a potential antifibrotic target and a few clinical studies addressing this have been performed. A monoclonal antibody towards the integrin alphaVbeta6 (αvβ6), expressed in epithelial cells, was recently investigated as a treatment option for IPF [131], as αvβ6 can activate latent TGF-β [132], but the study was halted due to safety concerns. Given the multifunctionality of TGF-β and its critical role in a range of physiological processes complete blocking of its activity could be associated with significant risk. Selective inhibition using 5-HT_2B_ receptor antagonists that interfere with certain TGF-β signaling pathways could represent a more specific and safer alternative to a complete blockage (Figure 3).

An ongoing clinical trial in phase III examines the autotaxin inhibitor (GLPG1690) that reduces lysophosphatidic acid as its mode of action in patients with IPF. Interestingly, lysophosphatidic acid plays a crucial role in platelet activation as it acts as a stimulator of platelet aggregation [133]. Another compound being investigated is the pentraxin 2 protein, showing high tolerability in IPF patients and early signs of persistent treatment efficacy [134]. Pentraxin 2 protein is a natural circulating protein with immune modulating entities affecting macrophage differentiation, attenuating profibrotic phenotypes [135]. Interestingly, 5-HT_2B_ receptor activation has been shown to modulate human macrophage polarization, promoting M2 macrophages representing a profibrotic phenotype with immunosuppressive and wound reparative characteristics. Changes in the expression of M2 genes, e.g., CCL18, have recently been linked to clinical responses on lung function in tocilizumab-treated patients with SSc [136]. In addition, nintedanib, in clinical use for IPF and SSc-ILD [137], inhibits M2 differentiation of human monocytes in vitro and reduces M2 macrophage counts in vivo [138]. Macrophages recruited to sites of injury express increased levels of TNF-α, while levels of TGF-β1 are increased at later stages of wound healing [139]. These temporal alterations in inflammatory and fibrotic mediators, orchestrated by the wound healing response, further emphasize the complex mechanistic role of 5-HT_2_ receptor activation during fibrosis development and optimal windows for effective treatments. The immune modulating properties of 5-HT_2B_ receptor antagonism proposes thus a beneficial effect in antifibrotic treatments, since chronic inflammatory processes and enhanced release of proinflammatory mediators contribute to tissue destruction and remodeling, also seen in patients with RA and SSc. Treatment with anti-inflammatory agents in patients with PF-ILDs with elements of pulmonary inflammation may serve to counteract the positive feedback loop created in the tissue niche with ongoing remodeling. However, the underlying and powerful driver of fibrosis is situated also in the ECM as a pathologically restructured lung-ECM directs cellular responses towards a persistent profibrotic activity [140]. In a recent clinical trial, patients with PF-ILD, not focusing on disease origin, were investigated in regard to the efficacy of nintedanib [141]. The study showed promising results in affecting the annual decline in FVC, further emphasizing converging disease mechanisms in PF-ILDs.

In conclusion, there is compelling evidence pointing toward converging pathways in the fibrotic development in PF-ILDs, whereby hampering 5-HT_2B_ receptors activity alleviates several key pathological features in IPF, RA and SSc.

## Abbreviations

ILD	Interstitial lung disease
PF	Progressive fibrosing
IPF	Idiopathic pulmonary fibrosis
5-HT	Serotonin, 5-hydroxytryptamine
SSc	Systemic sclerosis
RA	Rheumatoid arthritis
UIP	Usual interstitial pneumonia
HRCT	High-resolution computed tomography
FVC	Forced vital capacity
NSIP	Nonspecific interstitial pneumonia
lcSSc	Limited cutaneous SSc
dcSSc	Diffuse cutaneous SSc
ACPA	Anti-citrullinated protein antibody
CNS	Central nervous system
TGF-β1	Transforming growth factor
TPH	Tryptophan hydroxylase
IDO	Indoleamine 2,3-dioxygenase
TDO	Tryptophan 2,3-dioxygenase
SERT	Serotonin re-uptake transporter
GPCR	G-protein coupled receptors
PLC	Phospholipase C
IP_3_	Inositol 1,4,5-trisphosphate
PI3K	Phosphoinositide 3-kinase
PAI-1	Plasminogen activator inhibitor 1
ECM	Extracellular matrix
α-SMA	Alpha-smooth muscle actin
TNF-α	Tumor necrosis factor-alpha
PDGF	Platelet-derived growth factor
VEGF	Vascular endothelial growth factor
IL	Interleukin
PAH	Pulmonary arterial hypertension
vWF	von Willebrand factor
Tsk-1	Tight skin 1
αvβ6	alphaVbeta6
